# Supplementary material for: Drug susceptibility profiles and factors associated with non-tuberculous mycobacteria species circulating among patients diagnosed with pulmonary tuberculosis in Tanzania
Source: PLoS One. 2022 Mar 24;17(3):e0265358. doi: 10.1371/journal.pone.0265358 (PMC8947393; doi:10.1371/journal.pone.0265358)
Supplement: Attachment — Submitted filename: Response to Reviewers.docx [file pone.0265358.s002.docx]

Dear Academic Editor, PLOS ONE

Thank you for providing us with an opportunity to submit a revised draft of our manuscript titled “Drug susceptibility profiles of non-tuberculous mycobacteria species circulating among patients diagnosed with pulmonary tuberculosis in Tanzania”. We appreciate the time and effort that you and the reviewers have dedicated to providing your valuable feedback on our manuscript. We are grateful to the reviewers for their insightful comments on our paper. We have been able to incorporate changes to reflect most of the suggestions provided. We have highlighted the changes within the manuscript.

Here is a point-by-point response to the comments and concerns.

**General comments**

**Comment G1**

*Please ensure that your manuscript meets PLOS ONE's style requirements, including those for file naming. The PLOS ONE style templates can be found at* 
[*https://journals.plos.org/plosone/s/file?id=wjVg/PLOSOne_formatting_sample_main_body.pdf*](https://journals.plos.org/plosone/s/file?id=wjVg/PLOSOne_formatting_sample_main_body.pdf) *and* 
[*https://journals.plos.org/plosone/s/file?id=ba62/PLOSOne_formatting_sample_title_authors_affiliations.pdf*](https://journals.plos.org/plosone/s/file?id=ba62/PLOSOne_formatting_sample_title_authors_affiliations.pdf)

**Response G1**

The entire manuscript has been revised to meet PLOS ONE's style requirements as specified in the shared template

**Comment G2**

*Please provide additional details regarding participant consent. In the ethics statement in the Methods and online submission information, please ensure that you have specified (1) whether consent was informed and (2) what type you obtained (for instance, written or verbal, and if verbal, how it was documented and witnessed). If your study included minors, state whether you obtained consent from parents or guardians. If the need for consent was waived by the ethics committee, please include this information*

*If you are reporting a retrospective study of medical records or archived samples, please ensure that you have discussed whether all data were fully anonymized before you accessed them and/or whether* *the IRB or ethics committee waived the requirement for informed consent. If patients provided informed written consent to have data from their medical records used in research, please include this information.*

**Response G1**

We have revised the “Ethical clearance” section of the manuscript to address the consent of the study subjects. As this study used archived samples, the need for consent was waived by the IRB, we have included a statement to the effect that “The need for consent for this study was waived by the ethical committee” and a sentence on ensured anonymity of the data has been added.

**Comment G3**

*Thank you for stating the following in the Acknowledgments Section of your manuscript: 
The research for this paper was carried out within the framework of the DELTAS Africa Initiative [Afrique One-ASPIRE /DEL-15-008]. Afrique One-ASPIRE is funded by a consortium of donors including the African Academy of Sciences (AAS) Alliance for Accelerating Excellence in Science in Africa (AESA), the New Partnership for Africa’s Development Planning and Coordinating (NEPAD) Agency, the Wellcome Trust [107753/A/15/Z] and the UK government.*

*We note that you have provided funding information that is not currently declared in your Funding Statement. However, funding information should not appear in the Acknowledgments section or other areas of your manuscript. We will only publish funding information present in the Funding Statement section of the online submission form. 
Please remove any funding-related text from the manuscript and let us know how you would like to update your Funding Statement. Currently, your Funding Statement reads as follows: 
The research for this paper was carried out within the framework of the DELTAS Africa Initiative [Afrique One-ASPIRE /DEL-15-008]. Afrique One-ASPIRE is funded by a consortium of donors including the African Academy of Sciences (AAS) Alliance for Accelerating Excellence in Science in Africa (AESA), the New Partnership for Africa’s Development Planning and Coordinating (NEPAD) Agency, the Wellcome Trust [107753/A/15/Z] and the UK government.*

*The funders had no role in study design, data collection, and analysis, decision to publish, or preparation of the manuscript.*

*Please include your amended statements within your cover letter; we will change the online submission form on your behalf.*

**Response G3**

We have revised the manuscript, removed funding information, rewritten the acknowledgment part and it now reads “We are grateful to the management of the Central Tuberculosis Reference Laboratory (CTRL) in Tanzania for accepting and giving permission on the use of the archived samples. Moreover, to the laboratory staff for support they gave during retrieving the archived samples, DNA extraction and genotyping. Also, we acknowledge the CTRL data management team who assisted in accessing the patients’ data and in analysis. Lastly but not least we acknowledge the TB patients whose samples were used in this analysis”

”.

**Comment G4**

*We note that you have stated that you will provide repository information for your data at acceptance. Should your manuscript be accepted for publication, we will hold it until you provide the relevant accession numbers or DOIs necessary to access your data. If you wish to make changes to your Data Availability statement, please describe these changes in your cover letter and we will update your Data Availability statement to reflect the information you provide*

**Response G4**

This is agreed upon and thank you. The data will be shared with the journal during resubmission of the manuscript as part of supporting information

**Editor’s additional Comment**

*Major Revision*

**Response G4**

Major revision has been carried out for the manuscript

**Reviewers' comments:**

Reviewer's Responses to Questions

*1. Is the manuscript technically sound, and do the data support the conclusions?

The manuscript must describe a technically sound piece of scientific research with data that supports the conclusions. Experiments must have been conducted rigorously, with appropriate controls, replication, and sample sizes. The conclusions must be drawn appropriately based on the data presented.*

*Reviewer #1: Yes*

**Response R1**

We are thankful for this compliment

*Reviewer #2: No*

**Response R2**

We have revised statements on testing controls, sample size and the conclusion so it can easily be understood. A statement on the use of validated test methods has been added and the use of DNAse as negative control has been corrected.

*2. Has the statistical analysis been performed appropriately and rigorously?*

*Reviewer #1: I Don't Know*

**Response R1**

We tried to report recovered species, we have provided drug susceptibility profiles, we have analyzed proportions, and measure of association between various host factors (like treatment history, HIV status, age, gender, etc) and the existence of the pathogen to determine the levels of significance.

*Reviewer #2: I Don't Know*

**Response R2**

We tried to report recovered species, we have provided drug susceptibility profiles, we have analyzed proportions, and measure of association between various host factors (like treatment history, HIV status, age, gender, etc) and the existence of the pathogen to determine the levels of significance.

*3. Have the authors made all data underlying the findings in their manuscript fully available?

The* [*PLOS Data policy*](http://www.plosone.org/static/policies.action#sharing) *requires authors to make all data underlying the findings described in their manuscript fully available without restriction, with rare exceptions (please refer to the Data Availability Statement in the manuscript PDF file). The data should be provided as part of the manuscript or its supporting information, or deposited to a public repository. For example, in addition to summary statistics, the data points behind means, medians, and variance measures should be available. If there are restrictions on publicly sharing data—e.g. participant privacy or use of data from a third party—those must be specified.*

*Reviewer #1: No*

**Response R1**

Data collected and used for analysis have been shared with this manuscript as supporting information

*Reviewer #2: No*

**Response R2**

Data collected and used for analysis have been shared with this manuscript as supporting information

*4. Is the manuscript presented in an intelligible fashion and written in standard English?

PLOS ONE does not copyedit accepted manuscripts, so the language in submitted articles must be clear, correct, and unambiguous. Any typographical or grammatical errors should be corrected at revision, so please note any specific errors here.*

*Reviewer #1: Yes*

**Response R1**

We are thankful for seeing strengths in this paper

*Reviewer #2: No*

**Response R2**

Several typographical or grammatical errors have been corrected

*5. Review Comments to the Author*

*Please use the space provided to explain your answers to the questions above. You may also include additional comments for the author, including concerns about dual publication, research ethics, or publication ethics. (Please upload your review as an attachment if it exceeds 20,000 characters)*

*Reviewer #1: In this study, authors have tried to identify Mycobacterium species in NTM-PD cases so that better treatment options can be done. There are a few concerns, under the following headings

1. Study sites
In 2018 a total of 27 201 TB cases were bacteriologically confirmed in (26)- Incomplete sentences*.

**Response** **R1**

The sentence was rephrased and completed and now reads “For 2018 a total of 27,201 cases of pulmonary TB were bacteriologically confirmed in Tanzania.

*2. Quality control
Is it DNA free water or DNAse free water?*

**Response R1**

The word has been changed from “DNA” to “DNAse”

*3. Factors associated with Non-tuberculous mycobacterial infection among patients with pulmonary TB
Similarly, this study found an association between NTM infection and age of patients (p
270 = 0.07367). - Elaborate*
**Response R1**

The statement has been changed and now reads “Most cases of NTM were in the reproductive age between 35 and 44 years which is similar to TB”

*4. Results Table 1 is not clear, not make any sense or give a better explanation in the results section*

**Response R1**

Thank you for pointing this out, however, we thought of a necessity to show the variation of banding pattern between the three techniques that led us to categorize the isolate into the specified species. With this having pointed out, it is easier for a reader to correlate between results and interpreted species. This table also shows the frequency at which each species was identified.

*5. Discussion Similarly to the González, SG (39) and contrary to Park SC and Dodd PJ (41,42) NTM were more frequently isolated from males when compared to females- Rephrase*

**Response R1**

The has been rephrased and now reads **“**In this study, NTM were more frequently isolated from males (twice) when compared to females. This is similar to what was reported by Gonzalez et al (39) but contrary to that reported by Park et al and Dodd et al (41,42)”

*6. Conclusion followed up by MABC (n=4); remove “up”*

**Response R1**

“up” has been removed and now reads “followed by MABC”

*Reviewer #2: Overall assessment: Major revision
The paper entitled " Drug susceptibility profiles of non-tuberculous mycobacteria species circulating among patients diagnosed with pulmonary tuberculosis in Tanzania" by Togolani Godfrey Maya et al., have tried to address an important issue of drug susceptibility associated with NTMs diagnosed with Pulmonary TB. It is well documented that many NTM) are saprophytic, however, several species of NTM have been shown to cause human diseases, Pulmonary NTM infections lead to TB-like disease called NTM pulmonary disease (NTM-PD). Due to variation in treatment options among NTM species, it is necessary to identify the species and determine drug susceptibility profiles to inform the choice of appropriate regimen for the disease. 188 isolates from TB patients diagnosed were screened for NTM and were genotyped to determine drug susceptibility profile to macrolide and aminoglycosides. Data suggest that out of all the isolates 24 were NTM of which 15 were M. avium sub. intracellulare 4 and a total of 10 isolates were MAC (n=6) and MABC (n=4). Further authors have shown that the MAC were the most frequently isolated NTM species followed by MABC. While all MAC and MABC identified, were susceptible to aminoglycosides. Although the rationale of this study is interesting it should be rejected because of lacks depth, clarity, and a possible explanation for mechanists, poor data presentation and


Comments:
1. Sample size in the study for NTM is too small. The majority of the NTM reported is in the HIV + cases (29%) as compared to HIV negative cases (10%). What could be the possible explanation?*

**Response R2**

We agree that the sample size was small, however, this study aimed only at identifying the existence of NTM of which its prevalence among patients presenting with TB symptoms is not yet well-established despite its recent increased impact on the management of TB. Of 188 mycobacteria isolates screened, only twenty-four (24) had/were NTM. This study provides evidence of the potential impact of NTM in the management of pulmonary infections including TB and sets the stage for further studies with a larger sample size.

Possible explanations on the high proportion of NTM among HIV-infected subjects have been provided in the discussion section.

*2. It would be better to include patients with no history of HIV infection. What about other immune comprised conditions. Dose these NTMs are also seen similarly.*

**Response R2**

It is reported in his study that out of 110 HIV negative patients, 11 had pulmonary NTM while out of 34 HIV positive patients 10 had pulmonary NTM co-infection. This study couldn’t include other conditions of compromised immunity because only data for HIV were available for the mycobacteria isolates studied. However, other studies have reported a high prevalence of NTM among patients with Chronic Obstructive Pulmonary Disease.

*3. No proper explanation of inclusion and exclusion criterion for including Pulmonary TB patients*

**Response R2**

A statement on inclusion and exclusion criteria used in this study has been inserted under the “study design and sampling” section of the manuscript says “Only pulmonary TB patients who were bacteriologically confirmed and their isolates available were included in this study. On the other hand, pulmonary TB patients who turned culture-negative or their isolates could not be found were excluded from this study”

*4. In the abstract it is written for 188 TB patients; however, data shown is for 144 HIV co-infected patients. Where 44 patients had no history of HIV????, if so, how was the status of NTM in these patients. There is no clarity in this regard???*

**Response R2**

In the results section of the main body of the manuscripts, 188 isolates were screened out of which only 144 were from patients whose HIV status was available. For the remaining 44, the HIV status was not indicated. Furthermore, in the results section three NTM isolates have been reported for which the patients’ HIV statuses were unknown.

*5. To do justice with the title of the manuscript, authors should include other TB conditions or change it as TB patients having HIV history.*

**Response R2**

Thank you for raising this suggestion. It would be interesting to include other TB conditions or change it to address TB/HIV patients however, we thought this could be out of the scope of our study. To make sure we are focused on addressing the agenda as appears in the title, we have revised this manuscript to report the characterization of non-tuberculous mycobacteria in terms of species, drugs susceptibility, and variation of these novel pathogens with other host factors that have been reported to similarly affect other *Mycobacteria tuberculosis* case management like treatment history and status, HIV status, gender, age groups, etc. Moreover, we have revised the title to **“Drug susceptibility profiles and factors associated with circulation of non-tuberculous mycobacteria species among patients diagnosed with pulmonary tuberculosis in Tanzania”** to capture the analysed factors associated with NTM infection.

*6. Figures are poorly represented*

**Response R2**

Figure 01 shows the characteristics of study subjects while figure 02 displayed characteristics of patients with NTM. We decided to use bar charts instead of pie charts for an easy visualization and clarity of the demographic data.

*7. Why M. avium was not used for the drug susceptibility profile test in Table 2?*

**Response R2**

If you refer to Table 2, the last row indicates drug susceptibility results of two *Mycobacterium avium* which were susceptible both to macrolides and aminoglycosides.

*8. Grammatical checks need to be done throughout the manuscript.*

**Response R2**

We have revised the entire manuscript for grammar correctness using the online Grammarly check application.

*6. PLOS authors have the option to publish the peer review history of their article (*[*what does this mean?*](https://journals.plos.org/plosone/s/editorial-and-peer-review-process#loc-peer-review-history)*). If published, this will include your full peer review and any attached files.

If you choose “no”, your identity will remain anonymous but your review may still be made public.*

***Do you want your identity to be public for this peer review?*** *For information about this choice, including consent withdrawal, please see our* [*Privacy Policy*](https://www.plos.org/privacy-policy)*.*

*Reviewer #1: No*

*Reviewer #2: No*

**Response**

I appreciate both the reviewers for providing their valuable time to give these comments which have significantly improved this work

We look forward to hearing from you in due time regarding our resubmission and we will be grateful to receive and further address your comments.

Sincerely,

Togolani Godfrey Maya 22/01/2022
